# Healthy school recognized campus: design and methodology of a hybrid type 2 implementation-effectiveness cluster randomized trial

**DOI:** 10.1186/s13012-026-01487-2

**Published:** 2026-02-05

**Authors:** Allyson Schaefers, Cassandra M. Beattie, Gabrielli T. de Mello, Alisha George, Kendra Marstall, Julie Gardner, Jacob Szeszulski

**Affiliations:** 1 Institute for Advancing Health Through Agriculture (IHA), Texas A&M AgriLife Research, 17360 Coit Rd, Dallas, TX 75252 USA; 2 Texas 4-H Youth Development, Texas A&M AgriLife Extension, 1470 William D Fitch Parkway, College Station, TX 77845 USA

**Keywords:** Body mass index, Exercise, Nutrition, Adolescent health, Health education, Cooperative extension, Implementation strategy, Obesity

## Abstract

**Background:**

About one-third of U.S. youth are overweight and most have at least one risk factor that increases their chance of developing cardiovascular or other chronic diseases. School- and research-based physical activity and healthy eating programs can reduce obesity and improve health outcomes; however, schools face many implementation challenges. Healthy School Recognized Campus (HSRC) bundles school- and researched-based programs to improve their implementation and student health outcomes. This paper describes the protocol for a hybrid type 2 implementation-effectiveness, cluster dual randomized controlled trial that evaluates the (aim 1) effectiveness of the HSRC initiative for improving health behaviors and (aim 2) the impact of an implementation strategy – school-to-school mentoring – on HSRC’s delivery.

**Methods:**

Students in 4th through 9th grade (*n* = 500) at public schools (*n* = 20) across East and Central Texas will be randomized at the school level to determine the effectiveness of HSRC (vs. waitlist control condition) on BMI z-score (primary outcome), physical activity measured via accelerometer, and skin carotenoids (i.e., fruit and vegetable intake; secondary outcomes). Assessments will occur at the start and end of one school year. Program implementers (*n* = 200) at schools will be randomized to assess the impact of the school-to-school mentoring strategy (vs. standard implementation) on HSRC’s acceptability, appropriateness, and feasibility (co-primary outcomes). Assessments will occur at the start, middle, and end of one school year. The assessment at the end of the school year will also include a concurrent mixed-methods approach (QUAL + QUAN), guided by the Consolidated Framework for Implementation Research (CFIR), to evaluate the school-to-school mentoring strategy. For quantitative outcomes, a generalized linear model framework will be used to evaluate HSRC and the school-to-school mentoring strategy.

**Discussion:**

This study’s innovative dual randomized design allows for rigorous assessment of HSRC on effectiveness outcomes and the evaluation of a school-to-school mentoring implementation strategy on implementation outcomes. If both HSRC and the school-to-school mentoring strategy have their hypothesized effect, we will be well positioned to address cardiovascular and other chronic disease risk factors among youth using a scalable, widely used approach within one of the largest health educator networks in the country.

**Trial registration:**

Clinicaltrials.gov on July 1, 2025 (NCT07079995).

**Supplementary Information:**

The online version contains supplementary material available at 10.1186/s13012-026-01487-2.

Contributions to literature

- This study advances implementation science through its novel use of a hybrid type 2, cluster dual randomized controlled trial, providing feedback about the acceptability and feasibility of this design in community settings.
- The school-to-school mentoring strategy was iteratively refined in partnership with Extension agents (i.e., community health educators), demonstrating how iterative refinement can lead to an implementation strategy that is ready to be evaluated via a clinical trial.
- Concurrent testing of an intervention and an implementation strategy via two clinical trials will provide rigorous evidence that can be used to further inform the use of Extension’s evidence-based health promotion programs.

## Background

Obesity rates among children and adolescents in the United States continue to climb, with nearly 1 in 5 children being classified as obese [1, 2]. Obesity has major impacts on a child’s health by affecting their cardiovascular and other chronic disease risk – often contributing to or creating additional comorbidities [3, 4]. Physical activity and healthy eating are critical behaviors for addressing obesity [5–7], and they provide additional physical and psychological health benefits (i.e., reduced anxiety and depression) [8–10]. However, recent data show that only about a quarter of children and adolescents meet the United States physical activity guidelines of 60 min per day, and physical activity has continued to decline over the past decade as sedentary behaviors and screen time have risen [11, 12]. Similarly, 49% of children have overall poor diet quality, which often consists of empty calories (e.g., soda, enriched grains), is high in sodium and sugar, and lacks fruits and vegetables [13, 14]. Research-based programs that effectively improve physical activity and healthy eating are valuable tools for addressing childhood obesity.

National public health agencies strongly recommend, and some states’ laws require, the delivery of one or more research-based programs within schools [15–17]. These programs are a common way that health educators aim to promote physical activity and healthy eating and reduce obesity among children and adolescents [15–17]. Schools are an ideal setting for early interventions, as they can establish healthy physical activity and dietary behaviors early in life [18]. Despite the substantial potential of schools for promoting health, critical barriers to implementing health promotion programs in schools persist, including staff-related challenges (e.g., negative attitudes, limited teacher time, insufficient training), student-related factors (e.g., low motivation, limited engagement), constrained resources (e.g., funding and personnel), and competing curricular priorities [19–22]. Addressing these barriers through implementation strategies – methods to enhance program delivery – is essential for translating research-based programs into meaningful health outcomes and for ensuring that programs are both feasible and scalable within the school setting [21, 23].

The Healthy School Recognized Campus (HSRC) initiative directly addresses these implementation challenges by offering a structured framework for schools to adopt and deliver multiple research-based physical activity and nutrition programs [24–27]. Rather than relying on a single curriculum, HSRC bundles complementary programs that collectively support healthier behaviors within the school environment [24–27]. Participating schools commit to implementing at least three research-based physical activity and/or nutrition programs, including: (1) an 8-week walking challenge [28–30], (2) at least one additional program for youth, and (3) at least one program for caregivers, such as parents and/or school staff. A defining strength of HSRC is its multilevel design that aligns with ecological frameworks for health [7, 31, 32]. More specifically, HSRC aims to help health educators who use it engage individuals (i.e., intrapersonal level), social networks (i.e., interpersonal level), and school support (i.e., organizational level) in delivering programs. Schools that complete HSRC also receive public acknowledgement via a banner and/or proclamations during school board meetings, which aligns with prior research showing that such recognition can support improvements in student health outcomes, including body mass index (BMI) [8].

Previous studies have evaluated the effects of the HSRC initiative on student health outcomes and the impact of implementation support strategies on HSRC’s delivery [24–27]. Preliminary data from a single-group pre- to post-test pilot study showed several promising, positive effects of HSRC on the physical activity, nutrition, and BMI z-scores of students [27]. However, a more rigorous study design is needed to better evaluate whether these outcomes are related to HSRC or to other factors occurring during the same period (e.g., the start of the school year) [33]. Similarly, previous work evaluating three different implementation strategies – additional incentives, engaging contests, and school-to-school mentoring – demonstrated that the school-to-school mentoring strategy was a promising approach to improving HSRC’s short-term implementation outcomes (e.g., acceptability, appropriateness, and feasibility) [24]. Nevertheless, it has not been evaluated compared to HSRC’s standard implementation process. As a result, additional rigorous randomized controlled trials are needed.

This paper describes the protocol for a hybrid type 2 implementation-effectiveness, cluster dual randomized controlled trial (DRCT). DRCTs include two randomized controlled trials embedded in the same study. The first embedded randomized controlled trial evaluates: (aim 1) the effectiveness of the HSRC initiative for improving health behaviors (physical activity and healthy eating) and outcomes (BMI z-score) compared to a control group. The second embedded randomized controlled trial evaluates: (aim 2) the impact of an implementation strategy – school-to-school mentoring – against standard implementation on implementation outcomes (acceptability, appropriateness, feasibility). Concurrently conducting two randomized controlled trials to address these aims represents an innovative approach to advancing research on effective school-based approaches to reduce obesity and other chronic disease risk factors.

## Methods

### Study setting and school recruitment

The Central and East regions of Texas have some of the highest rates of cardiovascular disease and stroke mortality in the United States [34]. Accordingly, we will recruit ten schools from these regions in year one and ten schools in year two to participate in this study. As HSRC is a Texas A&M AgriLife Extension initiative and given the Cooperative Extension systems’ ability to bridge the gap between universities and communities [35], we will work with AgriLife county Extension agents (i.e., health educators) to assist with recruiting and implementing programs in schools.

We will use Texas Education Agency data to identify public schools of similar size, rurality, socioeconomic status, and racial/ethnic composition that are either eligible for HSRC or have previously contacted AgriLife Extension expressing interest in participation [36]. Once schools are identified, a research staff member – with or without the help of a county Extension agent – will contact interested schools via phone call, email, informational presentations, and/or flyers to share information about the HSRC initiative and the study. Schools that express interest in participating will meet with the research team to review study expectations and procedures. If the school agrees to participate in the study, a school representative will sign a memorandum of understanding agreeing to participate and a site authorization to allow data collection at the school.

### Study design

**Fig. 1:**
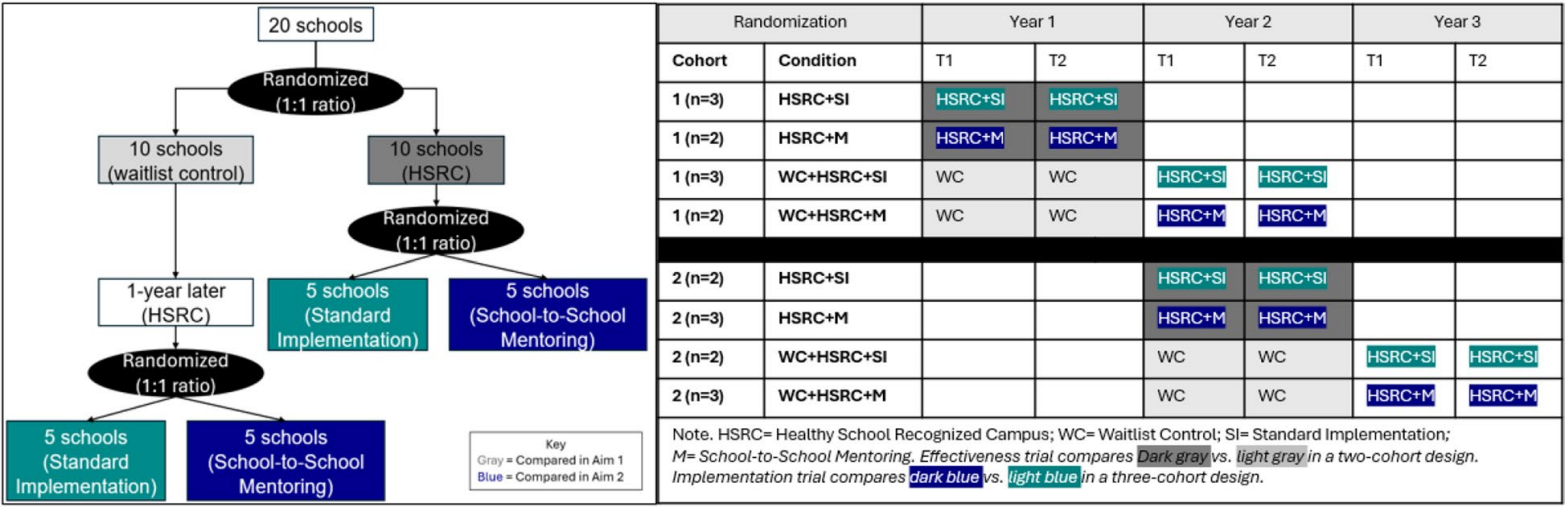
Randomization for the Cluster Dual-Randomized Controlled Trial

This study uses a hybrid type 2 implementation-effectiveness, cluster dual randomized controlled trial (DRCT; Fig. 1). DRCTs include two randomized controlled trials within the same study: one testing the intervention (i.e., HSRC) and one testing an implementation strategy [37–39]. DRCTs allow for rigorous testing of both the effectiveness of an intervention and the impact of an implementation strategy on outcomes, accelerating the translation of interventions into practice [37–39]. For the effectiveness aim (Aim 1), we will compare outcomes in ten Texas schools that receive HSRC with ten waitlist control schools (Fig. 1 in gray). For the implementation aim (Aim 2), ten schools will be randomized to receive a school-to-school mentoring program, and ten will receive standard implementation of the program (Fig. 1 in blue).

### Procedure

#### Randomization

Schools will be randomized to receive one of four conditions: (1) immediate intervention and standard implementation (HSRC + SI), (2) immediate intervention and school-to-school mentoring (HSRC + M), (3) waitlist control and standard implementation one year later (WC + HSRC + SI), or (4) waitlist control and school-to-school mentoring one year later (WC + HSRC + M). To ensure balance across years, schools will be blocked into two cohorts of ten schools, with five receiving immediate intervention and five receiving waitlist control in each of the first two years. Additionally, three schools will receive standard implementation in year one, five in year two, and two in year three, whereas two schools will receive school-to-school mentoring in year one, five in year two, and three in year three (Fig. 1). The order of the ten conditions available each year will be randomized, and schools will receive them based on the order in which they enroll in the study (i.e., signed memorandum of understanding).

#### Student eligibility and recruitment

Irrespective of the school’s randomized conditions, student recruitment will occur at the beginning of the school's first year of enrollment. Recruitment strategies will include both online (e.g., email) and in-person outreach (e.g., back-to-school events). To be eligible to participate, students must be enrolled in 4th through 9th grade, be between 8 and 16 years of age, and be able to speak, read, and write in English. Students with impairments (e.g., motor, cognitive) or health conditions (e.g., pregnant) that would prevent completion of study assessments will be excluded. Caregivers will complete a consent form and a brief demographic survey at baseline. To consent for the student, caregivers must be at least 18 years old. Students will provide in-person assent before assessments. Students who decline to participate in the study or who are not enrolled by their caregivers will still be encouraged to engage in HSRC programming.

#### HSRC implementer eligibility and recruitment

Extension agents will build an implementation team of 5–10 people to support the HSRC programs at their school (e.g., approval, planning, and/or facilitation of HSRC). The implementation team may include school administrators (e.g., principal, vice-principal, superintendent), classroom teachers, physical activity staff (e.g., physical education teachers), nutrition staff (e.g., school nutrition directors), parent-teacher organization members, volunteers, other Extension agents, student implementers, or any other role that supports HSRC programs (e.g., school health advisory council members). Once Extension agents have built their teams, they will provide the names and contact information to the research staff. The research staff will send study information and an enrollment invitation to all implementation team members, including the Extension agent who formed the team, via email. Adult implementers must be at least 18 years of age and able to speak, read, and write in English to consent. Youth implementers will need a caregiver at least 18 years old to sign a consent form, and they must be able to speak, read, and write in English to participate.

#### Aim 1 (Effectiveness) intervention and control conditions

Schools randomized to the intervention will receive the HSRC initiative immediately, which includes multiple research-based programs shown to improve physical activity, healthy eating, and weight-related outcomes (examples in Table 1). Over the past three years, HSRC has been adopted by more than 50 school campuses across Texas and has reached more than 15,000 students [26]. A detailed description of the HSRC initiative is published elsewhere [24]. Briefly, the HSRC initiative encourages schools to complete a schoolwide walking program, a second program for students, and a program for adults (e.g., teachers or caregivers); however, schools can complete more than three programs. To support the implementation of programs, this study provides each school with a $2,000 stipend, and upon completion, they receive public acknowledgement via a banner and/or proclamations during school board meetings. Schools randomized to the waitlist control condition will maintain their existing practices without adding or removing physical activity or healthy eating programs for students in the 4th through 9th grades. Additionally, they will receive the opportunity to participate in HSRC the following academic year as part of the implementation trial (aim 2).

**Table 1:** Examples of Healthy School Recognized Campus’ Evidence-based Programs

Program	Description	Evidence
**Youth**		
Walk Across Texas! (Youth) [28, 29]	An 8-week program designed to help youth across Texas establish the habit of regular physical activity using a fun and motivating team approach	- Doubled the number of steps taken over the course of the program - Increased the amount of time that students were active with their parents - Decreased BMI percentile
Learn Grow Eat & Go [28, 40]	10-week program that teaches students about gardening, healthy eating, and being active	- Increased the number of vegetables tasted, vegetable preferences, and nutrition knowledge - Decreased BMI percentile
Choose Health: Food, Fun & Fitness [41–43]	6-session program that encourages healthy eating and fitness through hands-on activities and experiential education	- Improved overall dietary intake, vegetable intake, fruit intake - Reduced soda/fast food intake - Read nutrition labels and share about healthy eating with families more often - More likely to try new food -Increased frequency of doing physical activities
Color Me Healthy [44, 45]	9-week curriculum extension that focuses on healthy eating and physical activity via coloring, hands-on learning, and music to help children engage with their senses	- Increased fruit and vegetable consumption - Increased students’ knowledge of healthy eating, knowledge about physical activity, and physical activity during the school day
Play Streets [46–48]	Series of 1-day events with various activity stations that promote safe, fun, and active play	- Encouraged physical activity participation - Provided a safe space for outdoor play - Fostered social interaction and community connection
**Adult**		
Walk Across Texas! (Adult) [49]	8-week program designed to help Texans establish the habit of regular physical activity using a fun and motivating team approach	- Increased number of miles walked per week - Decreased leisure time sitting
Cooking Well with Diabetes [50]	3 to 4 lesson workshops that build skills towards planning, preparing, and cooking healthy meals	- Increase fruit and vegetable intake and the use of healthy cooking methods (e.g., baking, broiling, grilling) - Decreased sugar-sweetened beverage intake
Maintain No Gain [51]	6-week holiday challenge to help adults maintain their current weight between Thanksgiving and New Years. Weekly weigh-ins and exercise challenges are provided	- A majority (~ 80%) of participants maintained or improved their weight status during the program

#### Aim 2 (Implementation) implementation strategy and control conditions

Schools randomized to receive the school-to-school mentoring strategy will participate in a series of activities designed to provide a support system, technical assistance, and facilitate connections between schools concurrently implementing HSRC, as a way to improve implementation outcomes (i.e., acceptability, appropriateness, feasibility). Immediately following randomization to the school-to-school mentoring strategy, participating schools will receive an introductory email welcoming them to the school-to-school mentoring program and informing them about its components (e.g., text messages, newsletters). The research team will host virtual meetings with implementers to discuss implementation successes and barriers. Schools will receive quarterly newsletters (up to four times during the year), which will include a previously recognized HSRC school, links to program implementation resources, a spotlighted HSRC program, and local county health-focused events. For agents who report two or more years of experience with HSRC, we will have them co-lead one monthly mentoring meeting and pair them with less-experienced agents to serve as HSRC mentors. Each month, we will send the Extension agent a reminder email and text message of how much of their $2,000 they have spent and how many programs they have completed. By reminding schools of how much money they have left and where they are in the process of getting the HSRC designation, we will continuously engage them with HSRC. Both the intervention and control groups (i.e., standard implementation) will have access to the HSRC website, which includes research evidence and details for each program, alignment of each program with Texas Essential Knowledge and Skills (TEKS) (i.e., curriculum standards for public schools in Texas), step-by-step instructions on how to earn the HSRC distinction, and additional resources.

#### Outcomes

Students will complete data collection two times over the course of the academic year. HSRC implementers will complete data collection three times over the course of the year. For both groups, baseline data collection will occur within the first four months (Aug. 1 – Nov. 30). For implementers, mid-point data collection will occur in the middle two months (January 1 – February 28). For both groups, post-test data collection will occur in the last two months (April 1 – May 31). Student data collection will include a survey, anthropometric assessments, and measures of physical activity and healthy eating at both time points. Students will receive an item worth $20 (e.g., a T-shirt or active toy) for each data collection time point. Data collection for implementers will include a survey at all three points and an in-depth interview post-intervention. Implementers will receive $15 for completing each survey and $40 for completing the interview.

##### Effectiveness outcomes

Our primary outcome for evaluating HSRC’s effectiveness (aim 1) is BMI z-score. Secondary outcomes include physical activity and fruit and vegetable intake. Other outcomes that we will measure include waist circumference, blood pressure, and cardiovascular fitness. Students will also complete a survey that includes sociodemographic characteristics (e.g., biological sex, age, grade level, race, and ethnicity).

- *BMI z-score (Primary)*. Height will be assessed with a portable Seca stadiometer to the nearest 0.5 inch, as the student is standing with feet together, head in the Frankfort plane, and arms hanging naturally at the sides. Weight will be measured using a Tanita body composition analyzer to the nearest 0.1 lb. We will complete measurements up to three times, and the two closest values will be averaged. Measurements will be converted to kilograms (kg) and meters (m), and BMI z-score will be calculated as kg/m2 and compared against normative values from the Centers for Disease Control and Prevention growth charts [52].
- *Physical Activity (Secondary)*. We will use the ActiGraph GT9X accelerometer (ActiGraph Corporation, Pensacola, Florida, USA) to assess physical activity. Students will be instructed to wear the accelerometer on their non-dominant wrist 24 h a day for seven consecutive days, and to remove the device only for showers or activities in which it could be submerged in water (e.g., swimming, bathing). Raw tri-axial accelerometer data (30 Hz) will be processed into estimates of time spent in light, moderate, or vigorous physical activity [53]. Daily physical activity will be calculated as the sum of all moderate-to-vigorous physical activity and standardized to an 8-h wear period [53]. Student data will be included in analyses if they have ≥ 3 monitoring days in which wear time is > 8 h [53].
- *Fruit and vegetable intake (Secondary)*. We will assess students’ fruit and vegetable intake using a Veggie Meter, a non-invasive portable device that measures skin carotenoid levels (i.e., a biomarker of fruit and vegetable intake) [54]. We will collect three readings per child, and the average of the three will be used for analysis.
- *Waist circumference (Other)*. Waist circumference will be measured using a portable measuring tape, containing a Gulick spring attachment, at the midpoint between the floating rib and the iliac crest [55]. Measurements will be conducted three times, and the two closest values will be averaged.
- *Blood pressure (Other)*. Blood pressure will be measured using an automated Omron sphygmomanometer [56]. Cuff size will be adjusted to match the student's upper right arm. Students will be seated for at least five minutes before the measurement. The measurement will be done up to three times, and the two closest values will be averaged.
- *Cardiovascular fitness (Other)*. Cardiovascular fitness will be assessed using the FitnessGram PACER (Progressive Aerobic Cardiovascular Endurance Run) test [57]. On this multi-stage shuttle run test, students run twenty meters back and forth to synchronized audio cues. The PACER is sequenced to begin at an initial running speed of 8.5 km/hour and progressively increase by 0.5 km/hour every minute thereafter [57]. A research team member will provide instructions to students in small groups before they complete the test. Cardiovascular fitness is then determined by using laps completed to estimate the maximum volume of oxygen the body can use during intense exercise (i.e., VO₂ max) [57].

##### Implementation outcomes

Primary outcomes for evaluating the school-to-school mentoring strategy (aim 2) are acceptability, appropriateness, and feasibility of the HSRC initiative, which we will measure using surveys and interviews. Other outcomes we will measure include the number of students reached and dose delivered. We will also ask implementers to complete a brief survey reporting their sociodemographic characteristics (e.g., biological sex, age, race, ethnicity, school affiliation, and years worked in education).

Acceptability, appropriateness, and feasibility of the HSRC initiative will be assessed among members of each school’s implementation team using three validated instruments: the Acceptability of Intervention Measure (AIM), the Intervention Appropriateness Measure (IAM), and the Feasibility of Intervention Measure (FIM) [58]. Each scale asks respondents to rate their level of agreement with statements about HSRC using a five-point Likert scale ranging from “completely disagree” to “completely agree.” For each measure, we will average item responses to produce a single score between 1 and 5, with higher values indicating more favorable perceptions of HSRC. Surveys will be administered before, during, and after HSRC, allowing us to examine changes in perceptions over time and compare differences between study conditions.

Within 4–6 weeks of the end of HSRC’s implementation, we will conduct interviews at each study school with the Extension agent (n = 20) and at least one other implementer (n = 20). We will use a semi-structured interview guide to ask implementers about the school-to-school mentoring strategy and the overall acceptability, appropriateness, and feasibility of HSRC. The interview guide will also address the five Consolidated Framework for Implementation Research (CFIR) domains – individual characteristics, innovation characteristics (e.g., the HSRC initiative), inner setting (e.g., school characteristics), outer setting (e.g., the community), and implementation process – to determine how the school-to-school mentoring strategy affected each of these domains [59, 60]. Interviews will be conducted in person or online, last 60–90 min, and be recorded.

To assess other implementation outcomes (number of students reached, dose delivered), implementers will be asked to track a variety of programmatic components, including which programs were delivered, the number of sessions delivered per program, the class size of each classroom receiving the selected programs, session attendance, and session duration. To evaluate the total number of students reached through HSRC programming, students will write their teacher's name on the student survey. We will determine dose delivered for participants in the study by using program metrics (number of sessions x session length) and summing them across the number of programs the students’ teachers received at the end of the school year.

### Analyses

#### Statistical analysis

For aim 1 (effectiveness), we will use univariate and bivariate statistics to determine the distribution of outcome measures and to identify relevant covariates [61]. To test intervention effects, students randomized to HSRC vs. the waitlist control group (independent variable) will be evaluated using a generalized linear model framework. Baseline scores will be added as a covariate, post-intervention scores as the outcome, and school as a clustering variable [62]. Other covariates (e.g., school size), identified in the preliminary bivariate analyses, will also be included in the model. If missing data occurs, we will mitigate potential biases by analyzing multiple imputed datasets under an intention-to-treat approach [63]. Analyses will be compared to per-protocol analyses [64].

For aim 2 (implementation), we will use a concurrent complementary mixed-methods approach to evaluate the impact of the school-to-school mentoring strategy on each implementation outcome [65]. Quantitatively, we will follow the same analysis approach described for aim 1. However, receiving the school-to-school mentoring strategy (yes vs. no) will be the independent variable, and implementation outcomes (e.g., acceptability, appropriateness, feasibility) will replace student outcomes.

Qualitatively, we will transcribe audio files from the interviews. Applying a directed content analysis and iterative categorization approach [66, 67], we will generate an a priori codebook that is based on our interview guide and the CFIR framework (two coders) [59, 60]. Queries will be performed for quotes assigned to each deductive code, then we will examine the relationship between the implementation strategy, barriers/facilitators, and implementation outcomes. As we are reviewing excerpts from the queries, we will note important findings and key points in ¼ −1-page written summaries. Finalized themes will be produced.

#### Power analysis

For aim 1 (effectiveness), our final sample will include 25 students per school (*n* = 20 schools; 500 students total). With 50% of students in the intervention group and 50% in the control group, using effects sizes, variance, and cluster effects based on prior research [27], calculations indicate 447 students are needed to achieve a power of 0.80 to detect group differences in BMI z-score. We will recruit 500 students to account for a 5% loss to follow up.

Given that we have made additional modifications to the school-to-school mentoring strategy, we consider aim 2 (implementation) to be a pilot cluster randomized controlled trial. Best practices suggest that pilot studies focus on logistics and refinement of implementation protocols, which we will iteratively address through this study [68]. Still, based on our expected recruitment of 20 schools with 8–10-person implementation teams, using a beta of 0.8, a type I error rate of 0.05, and an intraclass correlation coefficient of 0.015–0.030, we are powered to detect a small-to moderate mean difference of 0.25—0.27 points on a 5-point scale (effect size of d = 0.45).

## Discussion

Overall, we will use a hybrid type 2 implementation-effectiveness cluster DRCT to evaluate the effectiveness of the HSRC initiative on BMI z-scores and the impact of the school-to-school mentoring strategy on implementation outcomes. The HSRC initiative bundles multiple research-based physical activity and healthy eating programs to be delivered by one of the largest Extension networks in the United States [24–27]. Given the high rates of cardiovascular and other chronic disease risk factors in Texas – about 10% of students have three risk factors (e.g., metabolic syndrome) – scalable school- and community-based approaches are needed to address them [27]. Texas A&M AgriLife Extension has 250 offices, 900 Extension agents (i.e., health educators), and a network of about 100,000 volunteers that can provide schools with the adequate support to deliver HSRC programs [69]. If the research-based programs that are included within HSRC can improve physical activity, healthy eating, and weight outcomes as well as, or better than, current school-based approaches, AgriLife Extension will be well-positioned to address cardiovascular and other chronic disease risk factors among youth across Texas.

Unlike one-size-fits-all implementation strategies [70], we iteratively developed and refined our school-to-school mentoring strategy in partnership with Extension agents to address their real-world barriers to implementation. Iterative development that follows structured processes – such as the multiphase optimizations strategy’s (MOST) preparation, optimization, and evaluation phased approach – have been shown to drive continuous improvement and foster adaptability of implementation strategies to various contexts [71, 72]. Previously, as part of our formative research (i.e., preparation), we surveyed and interviewed extension agents that were already delivering HSRC [26]. Through this work, we found that tangible resources enabled the implementation of HSRC, and that stakeholder buy-in, implementation climate, and learning climate were related to the acceptability, appropriateness, and feasibility of HSRC [26]. These findings ultimately led to the development of three implementation strategies. Subsequently, we conducted a cluster randomized factorial trial to optimize our implementation approach by evaluating the independent and combined (i.e., synergistic, antagonistic) impact of the three implementation strategies (incentives, contests, mentoring) on HSRC’s delivery [24, 72]. Preliminary findings from that study indicated that school-to-school mentoring (alone) was the best method for facilitating the implementation of HSRC, but that it still needed additional refinement. Building on these insights, the current study evaluates this refined strategy using a more rigorous, randomized, waitlist-controlled design. Results from this study can inform additional refinements, if needed, and better identify ways in which the school-to-school mentoring strategy supports agents and school stakeholders in the delivery of HSRC.

This study further advances implementation science through its use of a hybrid type 2, cluster DRCT, which allows for simultaneous evaluation of both the effectiveness of the intervention (i.e., HSRC) and the impact of the implementation strategy (i.e., school-to-school mentoring) on outcomes [37–39]. By randomizing schools to HSRC or waitlist control, and to different implementation conditions, this study provides a unique opportunity to link improvements in HSRC’s implementation to changes in students’ health behaviors and outcomes. Additionally, the use of randomization to answer both implementation and effectiveness questions reduces selection bias, balances measured and unmeasured confounders across schools, and enhances the validity of causal inferences within both trials [73]. Furthermore, as both aims have adequate statistical power to detect small-to-moderate effects, our design makes efficient use of resources to conduct two randomized trials answering important questions that can help to close the research-to-practice gap.

A recent review identified three previous trials that used a DRCT design, none of which have published results, and none conducted in community-based settings [37–39]. Consequently, our study presents the first use of the DRCT within schools and within an Extension network. The DRCT was chosen not only for its scientific rigor, but also to help with some of the common challenges that occur when conducting research in communities. For example, control groups in effectiveness trials often receive no intervention or subpar implementation of the intervention (e.g., less attention) after serving as a control (i.e., waitlist control). In our study the waitlist control group will be randomized to the enhanced implementation strategy or standard implementation helping to ensure that schools in the waitlist control condition receive the same level of implementation support as schools that were randomized to receive the intervention immediately. Similarly, a delay in time to receiving an intervention (i.e., waiting one year) has the potential to affect implementation of that intervention. The DRCT balances both implementation strategy groups (enhanced vs standard) on whether they receive the intervention immediately or must wait one year, balancing this potential confounder across condition reduces this potential bias better than other types of designs often used in the community setting (e.g., step-wedge design). Consequently, findings from our trial will help to identify potential benefits and barriers to using the DRCT design in community settings, which can inform future research efforts.

Our mixed-methods approach to evaluating the school-to-school mentoring strategy strengthens this study’s contributions to the literature on implementation strategies. A mixed-methods approach will provide more comprehensive data than either qualitative or quantitative approaches alone, and it enables a greater understanding of multiple implementer experiences [65]. In this case, our mixed methods approach will help us to better understand the potential mechanisms by which the school-to-school mentoring program affects implementation outcomes, it provides insight into how and why the strategy works, or where challenges persist, and it will inform how different roles within the HSRC implementation team differentially experience the strategy. This level of detail in understanding how the school-to-school mentoring strategy works will help us to tailor the strategy to distinct roles in the future. Furthermore, our mixed methods approach enables us to contextualize quantitative results, identify necessary adaptations, and determine whether the strategy is feasible and sustainable across diverse contexts [65]. For example, components of the enhanced implementation strategy, such as the newsletter, text messages, and virtual meetings with implementers, may be transferable to other community-based delivery settings where physical activity and nutrition programs are delivered (e.g., recreation centers, churches, workplaces). If HSRC is effective at improving health behaviors and outcomes, and the school-to-school mentoring strategy improves implementation as we hypothesize, HSRC and the school-to-school mentoring strategy will be ready for scaleup statewide and/or adapted for use with other Extension networks across the United States. Consequently, the results from this study will help develop a foundation that can be utilized to prepare a fully powered, hybrid type 3 implementation-effectiveness or a dissemination trial [71].

## Conclusion

Using a hybrid type 2 implementation-effectiveness framework, this study employs a DRCT design to evaluate both the HSRC initiative and a refined implementation strategy (i.e., school-to-school mentoring). The first embedded trial examines the effectiveness of the HSRC initiative on student obesity, physical activity levels, and skin carotenoid levels. The second embedded trial examines whether the school-to-school mentoring strategy enhances the delivery of HSRC. Findings from the qualitative and quantitative data collected during this study will inform future school-based intervention design, provide practical guidance for implementing school- and research-based health programs, and lay the groundwork for expanding HSRC to schools nationwide.

## Supplementary Information

Additional file 1. Consort Checklist and Extensions for Cluster and Factorial Designs

Additional file 2. Standards for Reporting Implementation Studies: the StaRI checklist

## Data Availability

No datasets were generated or analysed during the current study.

## Abbreviations

AIM: Acceptability of Intervention Measure
BMI: Body Mass Index
CFIR: Consolidated Framework for Implementation Research
DRCT: Dual Randomized Control Trial
FIM: Feasibility of Intervention Measure
HSRC: Healthy School Recognized Campus
IAM: Intervention Appropriateness Measure
ICC: Intraclass Correlation Coefficient
PACER: Progressive Aerobic Cardiovascular Endurance Run
SI: Standard Implementation
TEKS: Texas Essential Knowledge and Skills
WC: Waitlist Control
