# Supplementary material for: Healthy school recognized campus: design and methodology of a hybrid type 2 implementation-effectiveness cluster randomized trial
Source: Implement Sci. 2026 Feb 5;21:19. doi: 10.1186/s13012-026-01487-2 (PMC12977675; doi:10.1186/s13012-026-01487-2)
Supplement: Supplementary file 1 — Additional file 1. Consort Checklist and Extensions for Cluster and Factorial Designs [file 13012_2026_1487_MOESM1_ESM.docx]

**Additional File 1:** Consort Checklist and Extensions for Cluster and Factorial Designs

| Item # | CONSORT checklist item | Extension for cluster designs | Page  No* |
| --- | --- | --- | --- |
| **Title and Abstract** | | | |
| 1a |  | Identification as a cluster randomized trial in the title | 1 |
| 1b | Structured summary of trial design, methods, results, and conclusions |  | 2-3 |
| **Introduction** | | | |
| 2a | Scientific background and explanation of rationale | Rationale for using a cluster design | 4-6 |
| 2b | Specific objectives or hypotheses | Whether objectives pertain to the cluster level, the individual participant level, or both | 6 |
| **Methods (Trial Design)** | | | |
| 3 | Description of trial design (such as parallel, factorial) including allocation ratio | Definition of cluster and description of how the design features apply to the cluster | 7-8 |
| 3b | Important changes to methods after trial commencement (such as eligibility criteria), with reasons |  | N/A |
| **Methods (Participants)** | | | |
| 4a | Eligibility criteria for participants | Eligibility criteria for clusters | 8-9 |
| 4b | Settings and locations where the data were collected |  | 6-7 |
| **Methods (Interventions)** | | | |
| 5 | The interventions for each group with sufficient details to allow replication, including how and when they were actually administered | Whether interventions pertain to the cluster level, the individual participant level, or both | 9-10 |
| **Methods (Outcomes)** | | | |
| 6a | Completely defined prespecified primary and secondary outcome measures, including how and when they were assessed | Whether outcome measures pertain to the cluster level, the individual participant level, or both (cassy – cite analysis) | 10-14 |
| 6b | Any changes to trial outcomes after the trial commenced, with reasons |  | n/a |
| **Methods (Sample Size)** | | | |
| 7a | How sample size was determined | Method of calculation, number of clusters(s) (and whether equal or unequal cluster sizes are assumed),  cluster size, a coefficient of intracluster correlation (ICC or k), and an indication of its uncertainty | 15 |
| 7b | When applicable, explanation of any interim analyses and stopping guidelines |  | n/a |
| **Methods (Randomization)** | | | |
| 8a | Method used to generate the random allocation sequence |  | 7-8 |
| 8b | Type of randomization;  and block size) | details of any restriction (such as blocking Details of stratification or matching if used | 7-8 |
| **Methods (allocation concealment mechanism)** | | | |
| 9 | Mechanism used to implement the random allocation sequence  (such as sequentially numbered containers), describing any steps  taken to conceal the sequence until interventions were assigned | Specification that allocation was based on clusters rather than individuals and whether allocation  concealment (if any) was at the cluster level, the individual participant level, or both | 7-8 |
| **Methods (Randomization Implementation)** | | | |
| 10 | Who generated the random allocation sequence, who enrolled participants, and who assigned participants to interventions | Replaced by 10a, 10b, and 10c |  |
| 10a |  | Who generated the random allocation sequence, who enrolled clusters, and who assigned clusters to interventions | 7-8 |
| 10b |  | Mechanism by which individual participants were included in clusters for the purposes of the trial (such as complete enumeration, random sampling) | 7-8 |
| 10c |  | From whom consent was sought (representatives of the cluster, or individual cluster members, or both) and whether consent was sought before or after  randomisation | 6-9 |
| **Method (Blinding)** | | | |
| 11a | If done, who was blinded after assignment to interventions (for example, participants, care providers, those assessing outcomes)  and how |  | n/a |
| 11b | If relevant, description of the similarity of interventions |  | n/a |
| **Methods (Statistical Methods)** | | | |
| 12a | Statistical methods used to compare groups for primary and secondary outcomes | How clustering was taken into account | 14-15 |
| 12b | Methods for additional analyses, such as subgroup analyses and adjusted analyses |  | n/a |
| **Results** | | | |
| 13-19 | Not Applicable (Protocol) | Not Applicable (Protocol) | n/a |
| **Discussion (limitations)** | | | |
| 20 | Trial limitations, addressing sources of potential bias, imprecision, and, if relevant, multiplicity of analyses |  | 15-18 |
| **Discussion (generalizability)** | | | |
| 21 | Generalisability (external validity, applicability) of the trial findings | Generalisability to clusters and/or individual participants (as relevant) | 15-18 |
| **Discussion (Interpretation)** | | | |
| 22 | Interpretation consistent with results, balancing benefits and harms, and considering other relevant evidence |  | 15-18 |
| **Other Information** | | | |
| 23 | Registration number and name of trial registry |  | Abstact, page 19 |
| 24 | Where the full trial protocol can be accessed, if available |  | Abstact, page 19 |
| 25 | Sources of funding and other support (such as supply of drugs), role of funders |  | page 19 |
